# Supplementary material for: Plant-Based, Antioxidant-Rich Snacks Elevate Plasma Antioxidant Ability and Alter Gut Bacterial Composition in Older Adults
Source: Nutrients. 2021 Oct 29;13(11):3872. doi: 10.3390/nu13113872 (PMC8624639; doi:10.3390/nu13113872)
Supplement: Supplementary file 1 [file nutrients-13-03872-s001.zip › nutrients-1389014-supplementary.pdf]

Supplement table S1 Ingredients, calories, and the contents of macronutrients and dietary fiber of the four plant-based smoothies

Smoothie	Ingredients	Calories (kcal)	Protein (g)	Lipids (g)	Carbohydrate (g)	Dietary fiber (g)
Dark green	Chinese kale<br>Cucumber<br>Pineapple<br>Apple<br>Cashew<br>Walnuts	148	4	7	21	2.9
Green	Chinese mustard<br>Cucumber<br>Pineapple<br>Apple<br>Cashew<br>Walnuts	145	4	7	21	2.7
Purple	Beetroot<br>Purple cabbage<br>Purple grapes<br>Pear<br>Cashew<br>Walnuts	169	5	7	24	4.3
Dark green	Carrot<br>banana<br>pineapple<br>cashew<br>walnuts	186	5	7	29	4.7
